# Alternative surgical approach for inflatable penile prosthesis removal

**DOI:** 10.1186/s12610-020-00104-6

**Published:** 2020-05-18

**Authors:** Abdalla Alhammadi, Maher Abdessater, Abdulmajeed Althobity, Anthony Kanbar, Walid Sleiman, Bertrand Guillonneau, Ahmed Zugail, Sebastien Beley

**Affiliations:** 1 Department of Urology, Groupe hospitalier Diaconesses-Croix Saint Simon, Paris, France; 2 Department of Urology and Renal Transplantation, APHP- La pitié Salpêtrière University Hospital, Paris, France; 3 Department of Urology, Clinique Turin, Paris, France; 4 Department of Urology, Centre Hospitalier René DUBOS, Pontoise, France

**Keywords:** Implants peniens hydrauliques, Retrait, Approche, Infection, Inflatable penile prosthesis, Removal, Approach, Infection

## Abstract

**Background:**

The Inflatable penile prostheses (IPP) are used as definitive treatment for severe erectile dysfunction. Removal of an IPP can be challenging, especially for the non-andrologists and junior urologists. The classic penoscrotal incision for explanation can disrupt anatomy, which increases the risk of complications and makes future re-implantation difficult. This article aims to describe a simple surgical method for the removal of IPP, which avoids the penoscrotal incision and reduces the risk of urethral damage and additional fibrosis.

**Material and methods:**

Between November 2015 and February 2019, 15 patients underwent IPP removal using the same technique. Multiple incisions were performed directly over each component of the IPP for their removal. Four incisions of 2 cm each were made at the following sites: one incision on both sides of the ventral base of the penis, one inguinal incision, and one scrotal incision. Each incision provides direct access to one component of the IPP (cylinders, reservoir, and pump).

**Results:**

The mean duration of the surgery was 41 min (between 35 and 48 min). All procedures were completed successfully with a smooth course. None of the patients had any residual component of the IPP at the time of surgery. Neither complications (urethral or intestinal injury) nor excessive bleeding (> 100 mL) were documented in all patients.

**Conclusion:**

Our approach provides direct exposure of all components of the IPP. It reduces the risk of urethral iatrogenic injury and the local fibrosis (which is greater with the penoscrotal incision) that may impair future reinsertion of IPP. It is simple, safe, reproducible and easy to be performed by junior or unexperienced urologists in urgent cases.

**Resume:**

**Contexte:**

Les implants péniens hydrauliques (IPH) constituent le traitement définitif des dysfonctions érectiles. Le retrait de ces prothèses peut être difficile, surtout pour les jeunes urologues. L’incision classique péno-scrotale est. peu anatomique. Elle est. associée à un risque de plaies urétrales et de fibrose pouvant limiter l’insertion ultérieure de nouvelles prothèses. Nous présentons dans cet article une méthode alternative simple pour le retrait des IPH qui permet d’éviter l’incision péno-scrotale et les risques qui s’y associent.

**Matériel et Méthodes:**

Entre novembre 2015 et février 2019, 15 patients ont été opérés pour extraction d’IPH infectés, en utilisant la même technique chirurgicale. Une incision de 2 cm est. réalisée en face de chaque élément de l’IPH pour permettre son extraction: une incision scrotale, une autre inguinale et une de chaque côté de la base ventrale du pénis.

**Résultats:**

La durée moyenne de l’intervention était de 41 min (entre 35 et 48 min). Toutes les interventions ont été bien tolérées. Les éléments des IPH ont été retirés sans fragments résiduels. Aucune complication n’a été notée.

**Conclusion:**

L’approche décrite permet l’exposition directe des composantes des IPH. Elle permet la réduction du risque des lésions urétrales iatrogène et de fibrose ultérieure. Celle-ci est. plus fréquente avec l’incision péno-scrotale et peut limiter l’éventuelle insertion de nouveaux implants. Notre technique est. reproductible et simple pour être appliquée sans danger par les jeunes urologues peu expérimentés.

## Introduction

Erectile dysfunction (ED) is a disorder that affects 152 million men worldwide, and this number is estimated to reach 322 million by the year 2025 [1]. Inflatable penile prostheses (IPP) are used as a definitive treatment for severe ED after the failure of conservative medical treatment, or when the latter is contraindicated [2]. The implant is very effective with high levels of patient and partner satisfaction. Infection, hematoma, corporal fibrosis or perforation, erosion, urethral injury, and glandular ischemia are reported in 5% of patients after IPP implantation [3]. The rate of device mechanical failure is around 15% at 5 years. The infection of the device is rare (1–3%), but devastating when happening. The actual low rate of infection is the result of the use of antibiotic-coated devices, implementation of prophylactic antibiotic regimens, improvement of skin preparations, and the use of the “no-touch” technique during implantation [4]. In IPP infections, the removal of all the components of the device is recommended. The extraction of the reservoir is surgically challenging due to its anatomical location. The classic penoscrotal incision for explantation may lead to the injury of the urethra and the disruption of the anatomical structures and may damage the surrounding tissues, predisposing to fibrosis and making future re-implantation difficult [3].

We aim by this article, to describe a simple surgical technique for the removal of penile prostheses, that avoids the penoscrotal incision and its associated complications. It was developed by an expert andrological team with 10 years’ experience in the domain and became the standard technique at our institution.

## Material and methods

### Patients selection

Between November 2015 and February 2019, 15 patients underwent IPP removal using the same technique, by the same surgical team. Indications for removal were divided into infectious in 12 patients (80%), and non-infectious in 3 cases (20%) related to an unsatisfactory result, patient discomfort or device erosion. The former group had the prosthesis removed between 2 and 36 days from the onset of infection after the failure of conservative treatment. Voluntary removal of the device was performed 10 days after its implantation in the three patients. The demographic characteristics of the patients are detailed in Table 1. Two types of IPP were identified: Titan® Touch (Coloplast Group, Humlebaek, Denmark) in 11 patients (73%) and Titan® OTR (Coloplast Group, Humlebaek, Denmark) in 4 patients (27%). The causes of ED that lead to the implantation of the IPP are detailed in Table 2.

**Table 1:** Patients’ demographic data

Variable	Range	Mean
Age (years)	49–71	59.53
Body mass index (kg/m^2^)	21–35	28
Time between implantation and removal (months)	0.3–54	22.77

**Table 2:** Causes of erectile dysfunction leading to penile prosthesis insertion

Cause	N	Percentage
Radical prostatectomy	4	27%
Pelvic radiotherapy	3	20%
Diabetes mellitus	2	13%
Radical prostatectomy + Diabetes mellitus	2	13%
Radical cystoprostatectomy	2	13%
Radical prostatectomy + Pelvic radiotherapy	1	7%
Colectomy	1	7%
Total	15	100%

### Surgical technique

**Fig. 1:**
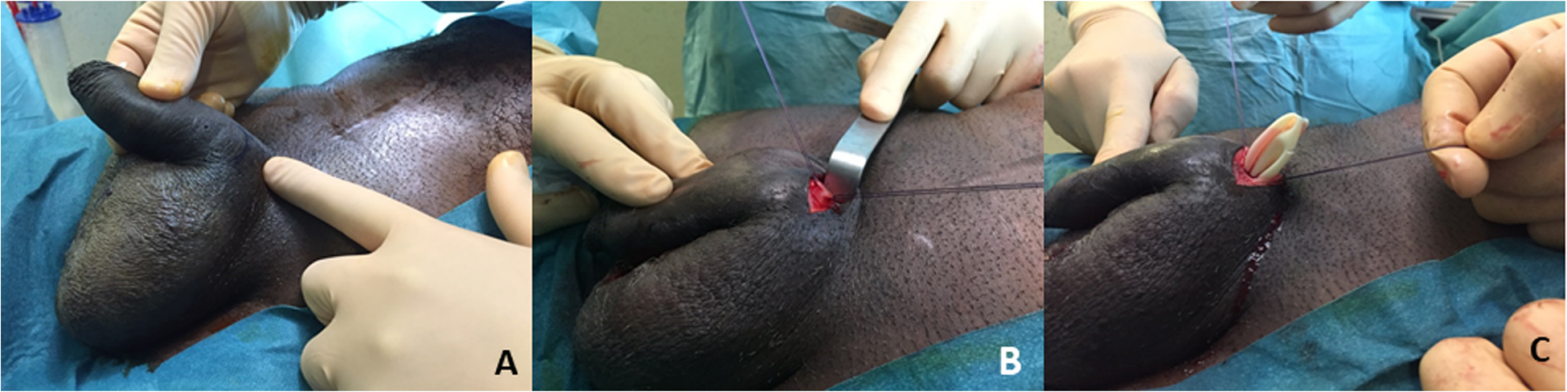
**a**- The incisional line drawn by a sterile surgical marker at the base of the penis. **b**- Stay suture on each side of corporotomy. **c**- The proximal part of the cylinder is delivered manually

**Fig. 2:**
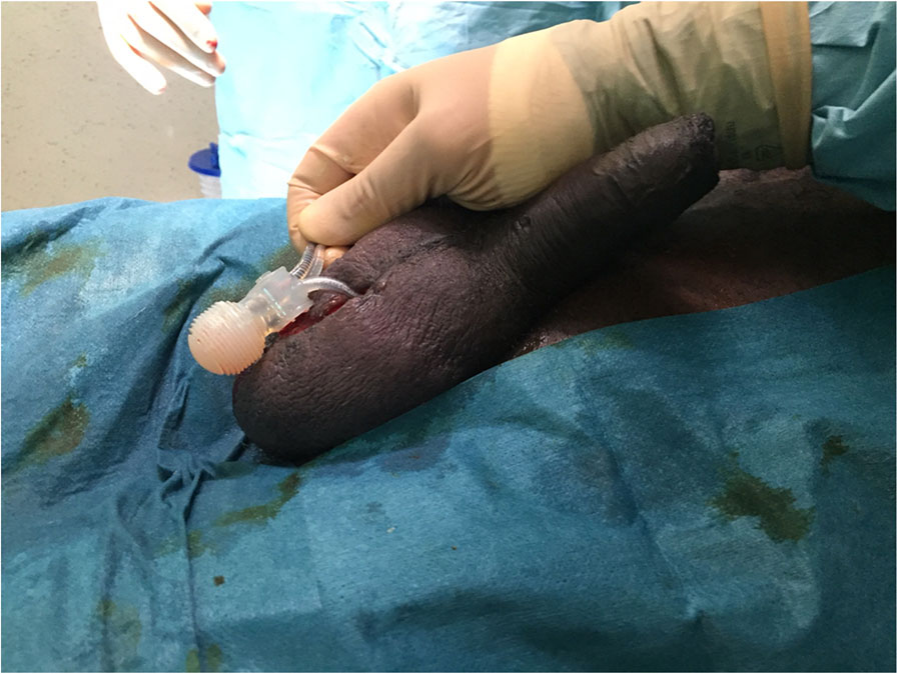
Extraction of the pump is done after incising the scrotum over it

**Fig. 3:**
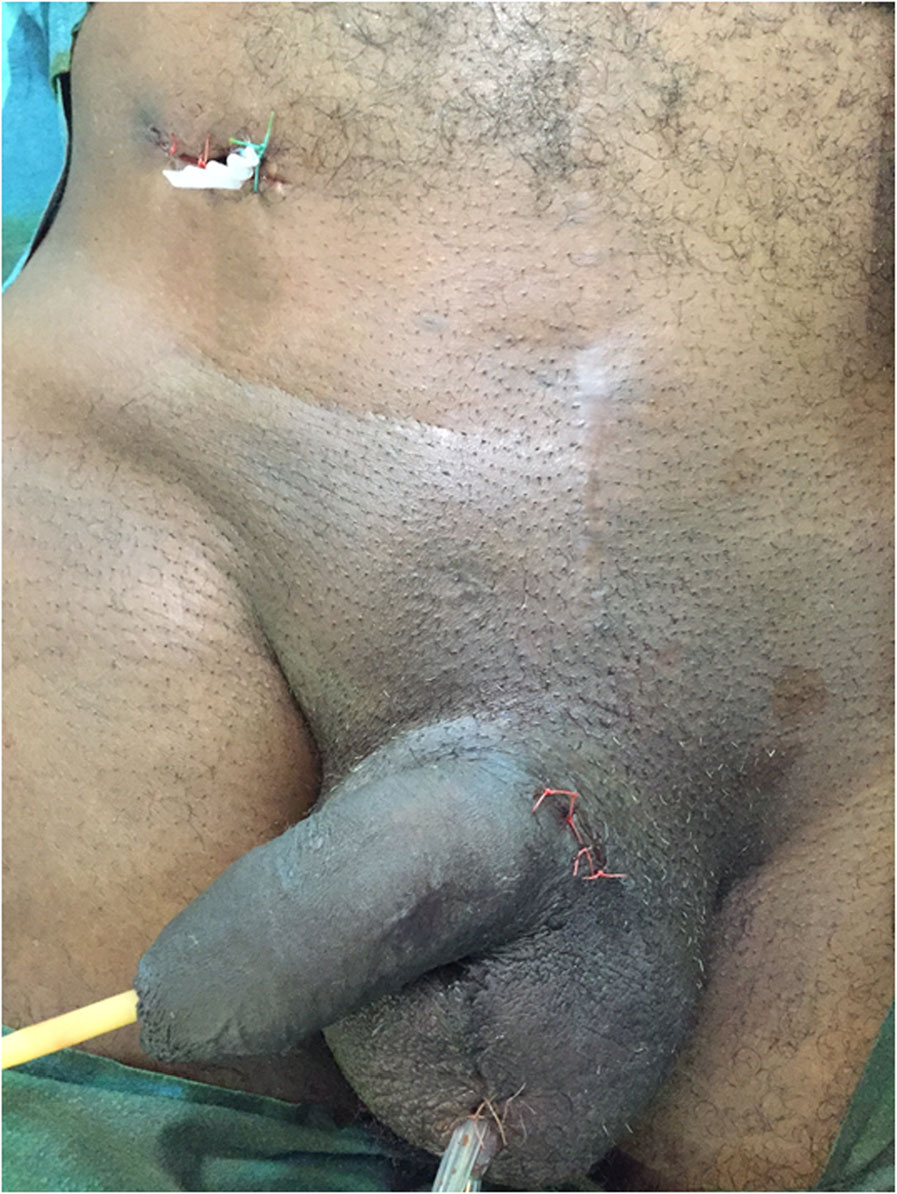
The final result of the procedure: the removal of the reservoir by an inguinal incision and the placement of a corrugated silicone sheet drain inside the inguinal and scrotal incisions that are fixed to the skin

After obtaining the patient’s consent, general or spinal anesthesia is applied. The patient is placed in a supine position, and an indwelling urinary catheter is inserted. The skin is shaved and prepped with an alcoholic-iodine solution. A 2 cm transverse incision is performed at each side at the ventral base of the penis (Fig. 1a). A corporotomy is done using a diathermy pencil, and a 2–0 absorbable stay suture is placed on each side of the corporotomy (Fig. 1b). The cylinders of the IPP are exposed and extracted using a Kelly clamp (Fig. 1c). Their rear tips are sent to the microbiology laboratory for culture. Another 2 cm scrotal incision is made directly over the pump. The optimal goal is to remove the pump with its pseudo-capsule (Fig. 2). A clamp is placed on the tube connecting to the reservoir and tugged to facilitate finding the reservoir. A transverse inguinal incision is carried out over the reservoir to allow its exposure and removal (Fig. 3). After the removal of all the components of the IPP, a culture swab is taken from the infected tissues. Before closing the wounds, tissues are irrigated with a mixture of iodine, hydrogen peroxide and normal saline using a 60 ml catheter tip syringe to wash out infected debris. Two corrugated silicone sheet drains (Delbet drains) are placed in the wounds: one in the inguinal incision and the other in the scrotal one. The drains are fixed to the skin using braided non-absorbable sutures. Finally, the corporotomy edges are closed using monofilament absorbable sutures and the skin is approximated using simple non-absorbable monofilament sutures.

### Post-operative management and follow-up

Broad-spectrum antibiotics are administered intravenously. We use amoxicillin with clavulanic acid or a fluoroquinolone depending on the patient’s associated risk factors. Pain management is provided during 2 to 3 days of hospitalization. Daily wound care is applied by the injection of a mixture of normal saline and iodine solution in the corrugated drains, followed by normal saline irrigation. The drain is mobilized exteriorly 1 to 2 cm each day starting from the second postoperative day and depending on the secretions. The previously described points are continued at home with a trained nurse whenever the infection is controlled and the patient is ready for discharge. After complete removal of the drains, open wounds at their corresponding sites are left to heal by secondary intention. A close follow up is necessary to examine the healing wounds and to adapt the antibiotics when necessary. The non-absorbable skin sutures are removed 7 to 10 days after the procedure. If distant re-implantation is anticipated, it is best done 2 to 3 months after the resolution of the infection, and the patient is given tadalafil 5 mg daily until the procedure.

We think that avoiding the bigger incision needed to remove all the components through the peno-scrotal incision leads to an easier reimplantation procedure, however we did not study the re-implantation results in our patients.

## Results

The duration of the surgery ranged between 35 and 48 min with a mean of 41 min. All procedures were completed successfully with a smooth course. None of the patients had any residual component of the IPP at the end of the surgery. Neither complications (urethral or intestinal injury) nor excessive bleeding (> 100 mL) were documented in all patients (Table 3). No significant more pain due to multiple incisions with this technique was reported by our patients.

**Table 3:** Outcomes of the described procedure

Technical details	Minutes
Minimum duration of the procedure	35
Maximum duration of the procedure	48
**Complications**	**N**
Residual component of the IPP	0
Urethral injury	0
Bleeding > 100 ml	0

## Discussion

Since the first IPP implantation described in 1973 by Scott et al., [5] many surgical techniques and devices have been described, progressively increasing patients’ safety and satisfaction [6]. Complications occur more commonly in patients with diabetes, spinal cord injury or immunosuppression [7].

After reviewing the English and French literature we found two papers describing techniques of IPP removal. Oesterling et al. described in 1989 the transurethral removal of eroded malleable prosthesis. After bringing its distal end into the fossa navicularis, the prosthesis is extracted through a transurethral incision of the corpora cavernosa [8]. Staller et al. published in 2016 the first article on the removal technique of infected IPP. The corporal cylinders and the pump were removed through a penoscrotal incision, while the reservoir was extracted using laparoscopic camera and instruments introduced through the same incision [9]. The transurethral removal is inappropriate in the case of infected IPP, because the healing of the iatrogenic caverno-urethral fistula will be impaired [10]. The endoscopic removal of the reservoir requires special skills for the use of the specific laparoscopic instruments and the three-dimensional spatial orientation, which are limitations in urgent septic cases that may be handled by junior urologists or surgeons with no or limited endoscopic experience. The classical penoscrotal incision is widely performed for the explantation of IPP. This approach offers great exposure and avoids dorsal nerve injury [11]. However, it carries a risk of iatrogenic urethral injury and makes the removal of the reservoir challenging in some cases. The local generated inflammatory response, the disruption of the surrounding tissues and change in the anatomical structures may predispose to fibrosis and make future implantations more difficult [3].

As the number of IPP procedures is increasing, there is a good chance that a non-experienced urologist will encounter the removal of an infected IPP in an urgent setting. The described procedure in this paper is fast and easy to learn. Every incision provides direct access to one component of the IPP, limiting the extension of the fibrosis and avoiding the centrally positioned urethra. It’s ideal for residents and junior urologists with little experience in andrology to manage infected IPP in urgent situations. Undoubtedly, the penoscrotal approach is advantageous when salvage re-implantation is considered since it offers better exposure of the corpora cavernosa.

## Conclusion

Our approach provides direct exposure of all components of the IPP. It reduces the risk of iatrogenic injury to the urethra and the local fibrosis (which is greater with the penoscrotal incision) that may impair future insertion of IPP. It is simple, safe, reproducible and easy to be performed by junior or unexperienced urologists in urgent cases. Further data and studies are required before the application of this technique as a standard method of removal of an IPP.

## Abbreviations

IPH: Implants peniens hydrauliques
ED: Erectile dysfunction
IPP: Inflatable penile prosthesis
